# Co-creating physical activity interventions: a mixed methods evaluation approach

**DOI:** 10.1186/s12961-021-00699-w

**Published:** 2021-03-15

**Authors:** Johanna Popp, Eva Grüne, Johannes Carl, Jana Semrau, Klaus Pfeifer

**Affiliations:** Department of Sport Science and Sport, Friedrich-Alexander University Erlangen-Nürnberg, Gebbertstraße 123b, 91058 Erlangen, Germany

**Keywords:** Coproduction, Cooperative planning, Participation, Pragmatic evaluation, Logic model, Health promotion, School, Workplace, Nursing care, Automotive mechatronics

## Abstract

**Background:**

Co-creation strategies, such as cooperative planning, are promising as a means to ensure that physical activity interventions address real-world problems and are tailored to the target group. This has already been validated in diverse settings. However, questions targeting the transferability of cooperative planning to new settings and the key factors influencing its success or failure remain unclear. At the same time, co-creation processes are complex, and evaluation can be challenging. Following calls for detailed reporting, this paper describes the programme activities, the underlying logic, and methodological design of a study that aims to evaluate the transfer of cooperative planning to new settings and to explore the associated key determinants.

**Methods:**

Cooperative planning was utilized as a strategy to target physical activity promotion in three real-world German settings in the nursing care and automotive mechatronics sectors. This involved researchers working alongside stakeholders from practice and policy to conjointly develop new interventions to promote physical activity in physically demanding jobs. A pragmatic approach is used to evaluate both the transferability and key determinants of this strategy. We developed a logic model for this co-creation process that describes the underlying assumptions and guides the evaluation. The evaluation outcomes of this study include planning meetings, newly developed interventions, and the determinants that are likely to affect cooperative planning. Quantitative and qualitative data will be collected using questionnaires, documents, and interviews. The quantitative data will be analysed descriptively, while the qualitative data will mainly be analysed using qualitative content analysis, split by settings. Subsequently, data triangulation will be used to integrate the quantitative and qualitative findings, which will then be compared across all three settings.

**Discussion:**

The study findings will contribute to a better understanding of co-creation strategies, their transferability, and key determinants. The practical implications can include a checklist for assessing key determinants and a guideline for transferring cooperative planning into new settings to benefit more people. Ultimately, this study will help to advance co-creation strategies and may be relevant for researchers, practitioners, and policy-makers targeting physical activity promotion in various contexts.

*Trial registration:* Open Science Framework: https://osf.io/r6xnt/ (retrospectively registered).

**Supplementary Information:**

The online version contains supplementary material available at 10.1186/s12961-021-00699-w.

## Background

Since the early years of health-related physical activity (PA) research [1], there has been a growing body of literature providing strong evidence regarding the beneficial effects of PA on an individual’s health [2, 3]. In accordance with the position that PA may work as medicine, a special focus should be put on population groups who are exposed to increased health risks and who may particularly benefit from the positive molecular and physiological effects of PA [4]. Even though work has entered the digital and automated era and a reduction in sedentary time has recently become a top priority in PA promotion [5–8], we must not lose sight of occupational groups who experience high physical demands during working life [9]. In line with the “settings for health” defined by the World Health Organization ([10], p. 362), work sites and schools appear to be favourable settings to promote health and PA by reaching people who work and learn there.

However, two aspects need to be considered in this context. First, when we aim to promote PA in professions with a high physical workload, the special demands of the occupational group must be taken into account. For example, there are indications that a high level of occupational PA is associated with a number of unfavourable health outcomes [11, 12]. In light of this, PA promotion among people with physically demanding jobs should not focus solely on increasing PA levels; the emphasis should be placed on strengthening competencies that are essential to master PAs in a healthy manner [13]. Second, the surrounding structures must be considered, as research has shown that supportive environments and policies are important for health behaviour changes [14, 15]. Taken together, it seems imperative to involve the target group and other nonacademic stakeholders as the experts in their settings [16–18] to identify appropriate PA interventions in physically demanding work settings.

Against this backdrop, co-creation, defined as “collaborative public health intervention development by academics working alongside other stakeholders” ([18], p. 2), is promising in terms of creating PA interventions. Involving different stakeholders in such strategies has the potential to systematically address real-world problems [19], to develop interventions that are tailored to the end user [18, 20], and to achieve sustainable outputs and impact [16, 21, 22]. The German research project PArC-AVE (Physical Activity-related Health Competence in Apprenticeship and Vocational Education) utilizes a co-creation strategy called cooperative planning (CP) [23] to develop interventions aimed to promote PA in the nursing care and automotive mechatronics sectors – two physically demanding professions. The goal of CP is to achieve changes at both the structural and individual levels that facilitate the adoption and/or maintenance of a physically active and healthy lifestyle; more specifically, by creating PA-friendly structures and strengthening Physical Activity-related Health Competence (PAHCO) [24, 25] among individuals. Previous research findings have shown promising results from CP in a private vocational education centre for health professions and in a vocational education centre of a German automotive manufacturer [26]. In both settings, new PA interventions were developed and implemented, and several capacities for PA promotion were built up. Thus far, it remains unclear whether CP can successfully be transferred to other settings to reach more people in the nursing care and automotive mechatronics sectors (in the sense of “scaling up” [27, 28]). Beyond that, the aforementioned study [26] identified differences between the nursing and automotive settings, mainly concerning the active participation of the target group and facilitators as well as barriers to intervention development and implementation (e.g. financial resources, organizational support). Although these differences have not been investigated in detail, we nevertheless note the importance of influencing factors, such as the attitudes of the involved stakeholders, support from organizations or individuals, and financial resources (see also [29]). This raises the question of which determinants are key enablers of and barriers to CP and thus need to be considered in future PA-promotion programmes.

These unanswered questions targeting the transferability and relevant determinants of CP gain more weight in light of the increasing body of studies using CP [30–33] or similar strategies in PA promotion and health promotion [22, 34, 35]; not forgetting the critical voices discussing the limitations and challenges of co-creation strategies [36, 37]. However, co-creating new interventions to promote PA is a complex process due to the high number of involved interest groups, the variability of outcomes, and the flexibility of the process [16, 38, 39]. Moreover, practical challenges for evaluation may arise from the real-world settings in which research in the PArC-AVE Project is conducted [40, 41]. Hence, this study uses a pragmatic approach, which seems favourable for the evaluation of complex programmes in real-world settings [41, 42]. We will build on the most relevant and best available data sources to gain a better understanding of how CP can be transferred to new settings and the factors linked to success or failure.

The current paper describes in detail the evaluation design of a study that aims to investigate the transferability of CP and to explore the key determinants for developing and implementing PA interventions. Following calls for more precise reporting of such studies (e.g. [29, 38, 43, 44]), we provide a thorough description of the activities that were undertaken, the underlying programme logic, and the evaluation design. Ultimately, this study will help to increase the knowledge base of co-creation strategies in the field of PA promotion and to foster their scientific as well as practical advancement.

## Methods

### Study settings and co-creation strategy

Building on the first research findings from 2015 to 2018 [26], the PArC-AVE Project aims at transferring CP as a co-creation strategy into three new settings in the nursing care and automotive mechatronics sectors from 2018 to 2021. The new settings are a state vocational education centre for health professions in a medium-sized city (setting A: 200 nursing students enrolled in the nursing programme), a state vocational education centre for health professions in a large city (setting B: 180 nursing students enrolled in the nursing programme), and the assembly department of a German automotive manufacturer (setting C: 12 000 employees in the assembly department), all located in Bavaria, Germany. In all three settings, the overall goal is to conjointly develop new PA interventions.

**Fig. 1:**
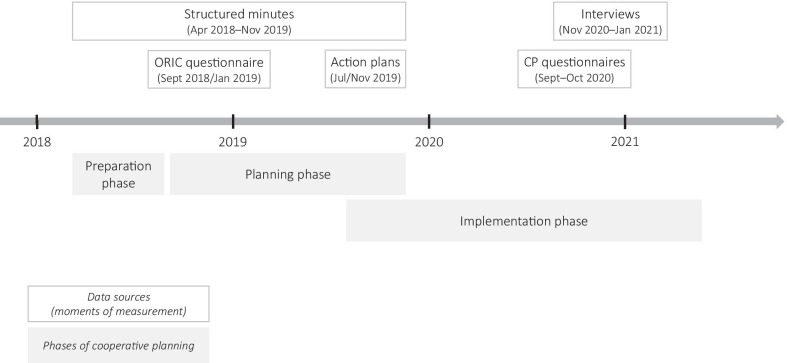
Overview of cooperative planning within the PArC-AVE Project (summary of settings A, B, and C). *CP* cooperative planning, *ORIC* Organizational Readiness for Implementing Change

Separate CP processes were undertaken in each setting (setting A, setting B, setting C), including the preparation, planning, and implementation phases [26, 45]. Figure 1 provides a summary overview of these processes. After the initial cooperation requests to settings A, B, and C (November 2016) and the subsequent positive funding decision (February 2018), the activities started in spring 2018. During the *preparation phase*, the initial meetings were used to inform the project partners about the project idea and to identify setting-specific structures, processes, and the relevant stakeholders (April/May 2018). A detailed explorative situation analysis was conducted in all settings to collect more information about the context and the needs of the target groups (April–July 2018). This was mainly done during several visits by the researchers to each setting and the utilization of a criteria catalogue, based on the ecological model of Bauman et al. [14] and on earlier findings [26], to collect the relevant information. In the *planning phase*, planning meetings were held to develop new PA interventions by a planning group of different stakeholders from research, practice, and policy (September 2018–December 2019). For further information on the phases and common rules of the planning meetings as well as the specific roles of the participants, see the description of earlier CP processes ([26], p. 1580–1581, Table 1). Finally, a set of new interventions was finalized in an action plan for each setting. In the *implementation phase*, the interventions were implemented under the guidance of the practitioners, starting after the last planning meetings (from July/November 2019).

### Logic model

**Fig. 2:**
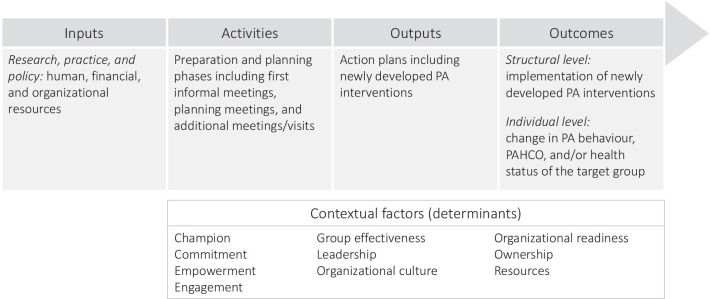
Logic model of the PArC-AVE Project. *PA* physical activity, *PAHCO* Physical Activity-related Health Competence

When evaluating such processes, logic models are a valuable first step, describing the potential mechanisms of how a programme works [46, 47]. Moreover, they have recently been identified as a potential framework for the evaluation of PA interventions in a systematic review by Fynn et al. [44]. In the present study, we developed a logic model to illustrate the planned activities and expected effects within the PArC-AVE Project and, in the next step, to guide the evaluation of CP and its determinants (Fig. 2). Usually, a logic model includes inputs, activities, outputs, and outcome components [47]; it can also include contextual factors that are expected to influence the success of the programme [48]. In our case, *inputs* can be defined as the resources provided by researchers, practitioners, and policy-makers to realize cooperation and planning meetings in a particular setting, such as human, financial, and organizational resources. *Activities* are all project meetings and visits in settings A, B, and C – most importantly, the planning meetings involving stakeholders from research, practice, and policy with the overall aim of developing PA interventions. *Outputs*, as the direct products of programme activities, are the developed interventions, documented in one action plan per setting. In accordance with the goal of CP including structural and individual changes, we differentiate between *outcomes* at the structural and individual levels. An outcome at the structural level is the implementation of the developed PA interventions; at the individual level, it is a change in the target group’s PA behaviour, PAHCO, and/or health status. *Contextual factors* are defined as the determinants that are likely to influence a CP process and its success or failure. Based on earlier findings within the project [26] and a literature screening of factors relevant to co-creation processes and the implementation of co-created interventions in January/February 2020, we identified the following determinants to be considered in the present evaluation: champion, commitment, empowerment, engagement, group effectiveness, leadership, organizational culture, organizational readiness, ownership, and resources. An overview of these predefined determinants and their definitions is provided as an additional file (see Additional file 1).

### Research questions and evaluation design

Two core research questions were identified to address the aim of this study:

Research question 1: How (un)successful is the transfer of cooperative planning to other settings?

Research question 2: Which key factors have an influence on the success or failure of cooperative planning?

The evaluation outcomes were subsequently derived from our logic model (see Table 1). To answer the first research question, planning meetings and the developed interventions will function as evaluation outcomes; measuring outcomes at the individual level (i.e. PA behaviour, PAHCO, health status) in a pre-post design was not possible in this study due to the early practitioner-initiated implementation of interventions. We will thus refer to the logic model components of *activities*, *outputs*, and *structural outcomes* to assess the success or failure of CP in settings A, B, and C. To answer the second research question, predefined and further determinants will function as evaluation outcomes, referring to the logic model component of *contextual factors*. Building on the differences between settings found in earlier research, we will contrast settings A, B, and C to examine potential differences and similarities. This will be followed by a final assessment of the success/failure of transferred CP and its relationships with the key determinants.

**Table 1:** Evaluation outcomes and data sources

Research questions	Evaluation outcomes *Logic model components*	Data sources	Participants invited
How (un)successful is the transfer of cooperative planning to other settings?	Planning meeting, i.e. number, organization, and realization of meetings, involved stakeholders *Activities*	Structured minutes (qual)	–
		CP questionnaires (quan)	All stakeholders who attended at least one planning meeting, excluding researchers
		Interviews (qual)	Main stakeholder(s) in each setting
	Developed interventions, i.e. number, content, and implementation status of interventions *Outputs and structural outcomes*	Action plans (qual)	–
		CP questionnaires (quan)	All stakeholders who attended at least one planning meeting, excluding researchers
Which key factors have an influence on the success or failure of cooperative planning?	Predefined determinants, i.e. level of impact *Contextual factors*	ORIC questionnaire [51] (quan)	All stakeholders who attended the first planning meeting, excluding researchers
		CP questionnaires (quan)	All stakeholders who attended at least one planning meeting, excluding researchers
		Interviews (qual)	Main stakeholder(s) in each setting
	Further determinants *Contextual factors*	Interviews (qual)	Main stakeholder(s) in each setting

*CP* cooperative planning, *ORIC* Organizational Readiness for Implementing Change, *qual* qualitative, *quan* quantitative

Inspired by the principles of a pragmatic evaluation [41], this study uses a mixed methods design to evaluate the transfer of CP and its determinants across three different settings [49]. Combining different methods and triangulating the quantitative and qualitative data will, finally, enable us to adequately answer both research questions [50].

### Data collection

The data will be collected using the following sources (quan = quantitative; qual = qualitative): questionnaires (quan), structured minutes (qual), action plans (qual), and interviews (qual). During the writing and submission process of this paper, the data collection already started. The moments of measurement are indicated for each data source in Fig. 1 and in the following text. An overview of the evaluation outcomes, data sources, and study participants split by research questions is depicted in Table 1.

#### Quantitative data

##### ORIC questionnaire

The predefined determinant of organizational readiness was assessed using the Organizational Readiness for Implementing Change (ORIC) questionnaire by Shea et al. [51], which has been shown to be a reliable and valid instrument of organizational readiness for change. This questionnaire contains 12 items using a five-point Likert scale to assess the domains of change commitment and change efficacy. As we could not find a German version during the preparation phase in 2018, we translated the questionnaire using forward and back translation with monolingual tests [52]. The forward translation was done by a native German speaker and the back translation was done by a native English speaker. Afterwards, three researchers validated and discussed both versions to refine the ORIC questionnaire in the German language. We also added an introductory description to explain the meaning of the term “change” in the given context, which in our case, are the changes at the organizational level concerning PA promotion that result from the research project. Yet, for future studies of organizational readiness in the German language, it should be noted that a German version of the ORIC questionnaire has recently been tested and published by Lindig et al. [53].

According to Weiner et al. [54], the assessment of organizational readiness should take place before the process of change begins. Utilizing a maximum variation sampling scheme [55], we asked all stakeholders who attended the first planning meeting in each setting, excluding the researchers, to participate in this paper-based survey before the meeting started (September 2018/January 2019).

##### CP questionnaires

One CP questionnaire per setting was developed to investigate (a) the organization and realization of planning meetings, (b) the implementation status of PA interventions, and (c) the impact of predefined determinants on CP.

- (a) We developed items assessing the contribution of different stakeholder groups to the planning meetings, the planning group’s structure and organization, the reasons for participation, and the satisfaction with CP. Most items were assessed using a five-point Likert scale, and some were assessed using a nominal scale or a dichotomous format.
- (b) For each PA intervention documented in the setting-specific action plan, items were formulated concerning the status of implementation as well as the fit and sustainability of the intervention. Items were assessed using either a nominal scale, a five-point Likert scale, or a dichotomous format.
- (c) We developed items for each predefined determinant (excluding organizational readiness, see the aforementioned ORIC questionnaire), based on their definitions and, if available, already existing instruments (see Additional file 1). Most items were assessed using a five-point Likert scale; only items concerning the determinant champion were assessed using a dichotomous format and open questions.

Given the restrictions due to the COVID-19 pandemic in 2020, we decided to conduct the CP questionnaires online using SoSci Survey (SoSci Survey GmbH, version 3.2.12, https://www.soscisurvey.de). In September 2020, all stakeholders who had attended at least one planning meeting, excluding the researchers, were contacted via email and asked to participate in the survey by following a setting-specific link (maximum variation sampling [55]).

#### Qualitative data

##### Documents: structured minutes and action plans

During the preparation and planning phases (April 2018–November 2019), structured minutes were taken of all meetings in the three settings. These contain information about the date and duration of the meetings, the participating stakeholders, and the main points raised during the discussions. At the end of the planning phase (July/November 2019), the newly developed interventions were finalized and described in one action plan per setting.

##### Interviews

Semi-structured interviews will be conceptualized to further assess key factors influencing CP, namely the development and implementation of interventions, and the way CP was used. In addition, the interviews will allow us to clarify potential ambiguities that may arise during the analysis of the questionnaires.

We will use setting-specific timelines to illustrate the entire CP processes from 2018 to 2021 and to support the temporal classification of influencing factors and moments. Timeline interviews are primarily used in life history research to analyse how life stories are related to the broader environmental, political, and social contexts [56], although they have already been employed to evaluate participatory research processes [57]. In the present evaluation, we will initially prepare one timeline template for each setting, drawing on information from structured minutes. These will include all meetings and further project-related activities in chronological order. Second, we will develop setting-specific interview guidelines by building on data collected via structured minutes, action plans, and questionnaires. The leading questions will target further influencing key factors, an overall appraisal of CP, and the clarification of inconclusive results from the questionnaires. Moreover, these questions will provide guidance to refine the setting-specific timelines, for example, by adding relevant key factors and moments that emerge during the interviews.

From November 2020 to January 2021, we will invite the main stakeholders in each setting via email to participate in an interview (purposeful sampling of information-rich cases [55]). One interview per participant will be conducted using online conference software, for example, Zoom (Zoom Video Communications, Inc.). Before the interviews start, the interviewees will be provided with written information about the study and will be asked to give their consent to participate.

### Data analysis

The collected data will be analysed for each data source separately, split by settings. Data triangulation will then be used to integrate the quantitative and qualitative findings in order to answer research questions 1 and 2 [50]. Subsequently, the findings will be compared across settings A, B, and C to identify potential differences and similarities.

#### Quantitative data

##### ORIC questionnaire

Data from the ORIC questionnaire will be analysed using SPSS Statistics (IBM). First, the items will be grouped according to the domains of change commitment and change efficacy. Second, the total ORIC, commitment, and efficacy scores will be calculated and analysed descriptively.

##### CP questionnaires

The setting-specific CP questionnaires will be analysed using SPSS Statistics (IBM). We will generate descriptive statistics to report characteristics of the planning meetings and the developed interventions as well as the impact of predefined determinants.

#### Qualitative data

##### Documents: structured minutes and action plans

Data from the structured minutes and action plans will be analysed descriptively by reporting the number and dates of the meetings, the number and characteristics of the involved stakeholders, and the number and content of PA interventions.

##### Interviews

All interviews will be audio-recorded and transcribed verbatim. For reasons of anonymity, we will substitute working positions for people’s names and pseudonyms for institutions and city names. The transcripts will be analysed using qualitative content analysis, involving a deductive and inductive definition of categories [58]. The data analysis will comprise the following steps: (1) initial text work, (2) deductive development of the main categories based on the interview guidelines, (3) coding of the entire material using the main categories, (4) compilation of all coded text passages with the same main category, (5) inductive definition of subcategories based on the transcribed material, (6) coding of the entire material using the refined categories, and (7) evaluation and interpretation [58]. MAXQDA (VERBI Gmbh) will be used for data coding and analysis. Two researchers will develop and double-check the main categories and subcategories and apply them to the interview transcripts. Inconsistencies will be discussed and resolved within the research team. The timelines created through the interviews will be pooled to generate one timeline per setting that will include the most important project activities and impactful factors and moments. Inconsistencies will be verified via data from structured minutes or general project documentation and, if necessary, clarified by interviewees or discussed in the team.

## Discussion

This methodological article describes the evaluation design of a study that aims to investigate a co-creation strategy – CP – in the field of PA promotion and, specifically, its transferability to new settings as well as the associated key determinants. CP was utilized in three real-world settings to develop and implement interventions targeting the promotion of PA and PAHCO among nursing students and employees working in an assembly department. Following recommendations to provide sufficient detail for such evaluation studies (see e.g. [29, 43]), this paper reports on the co-creation strategy, the underlying logic, and the evaluation design. Below, we outline the expected implications of this study.

First, the findings of this study will contribute to a better understanding of CP used as a strategy for promoting a physically active lifestyle. In particular, it will contribute to answering questions about the transferability or scaling up of co-creation strategies and the associated key determinants. Although co-creation in health promotion is neither a novel nor unknown topic in research, these are still relevant issues that are being discussed internationally (e.g. [22, 43, 59]). This pragmatic evaluation, emphasising relevance and practicability [41, 42], builds on a comprehensive data base and will, finally, enable us to increase the knowledge base of CP and similar strategies. In addition to our focus on school and occupational environments, the study findings might also be of interest for co-creation endeavours in other contexts, their further examination, and successful application.

From a practical point of view, recommendations for research, practice, and policy might be derived from the study results. For example, a checklist to systematically assess the key determinants of CP may help implementers decide whether more support (e.g. by leadership) or resources (e.g. time) are required even before collaboration starts. Another practical contribution could be the development of a guideline that supports the transfer of CP to further settings to reach more people (see scaling up [27, 28]). Taking into account recently published research findings, such as an initial guidance for research partnerships by Hoekstra et al. [34], a CP guideline might include all the necessary steps and helpful advices to successfully prepare, conduct, and evaluate a CP process. We think that both a checklist of key determinants and a guideline for CP could be useful tools for researchers in this field, but also for practitioners or policy-makers who are responsible for PA promotion (e.g. in schools, companies, communities).

In addition, we anticipate a substantial contribution to research examining strategies for promoting PA in physically demanding jobs. As described above, we hypothesise that this issue requires a special, target group- and setting-centred perspective. However, as the potential of CP as a suitable strategy has already been demonstrated elsewhere [26], our study will provide new scientific and practical insights and could even function as a starting point for further research in this field.

In summary, the present study will likely improve the understanding of how co-creation strategies can best be applied to address PA promotion in new settings. In doing so, it will add value to science and practice concerning the scaling up and advancement of such strategies. The results of this study may be relevant for co-creation and PA-promotion researchers, but also for people from practice and policy who deliver or make decisions about PA interventions.

## Supplementary Information

**Additional file 1: **Definitions and instruments underlying the operationalization of predefined determinants.

## Data Availability

The data sets generated and/or analysed during the current study, as well as the relevant research materials (e.g. questionnaires, interview guidelines), will be available from the corresponding author on reasonable request.

## Abbreviations

CP: cooperative planning
ORIC: Organizational Readiness for Implementing Change
PA: physical activity
PAHCO: Physical Activity-related Health Competence
PArC-AVE: Physical Activity-related Health Competence in Apprenticeship and Vocational Education
qual: qualitative
quan: quantitative
