# Supplementary material for: Co-creating physical activity interventions: a mixed methods evaluation approach
Source: Health Res Policy Syst. 2021 Mar 15;19:37. doi: 10.1186/s12961-021-00699-w (PMC7958094; doi:10.1186/s12961-021-00699-w)
Supplement: Supplementary file 1 — Additional file 1: Definitions and instruments underlying the operationalization of predefined determinants. [file 12961_2021_699_MOESM1_ESM.docx]

# Additional file 1

Definitions and instruments underlying the operationalization of pre-defined determinants

| **Determinant** | **Definition** | **Instrument** |
| --- | --- | --- |
| Champion | “a program champion who had advocated the continuation of the intervention was associated with an increased likelihood of sustainability” ([[1]](#_CTVL001176c085bf5084949b04d9c671206fa24), p. 707) | Survey instrument ([[1]](#_CTVL001176c085bf5084949b04d9c671206fa24), p. 710) |
| Commitment | “a volitional psychological bond reflecting dedication to and responsibility for a particular target” ([[2]](#_CTVL001de2c7c0b76d14f759a4748951bd04fe5), p. 137) | KUT (Klein et al., Unidimensional, Target-free) measure of commitment ([[3]](#_CTVL00163a9ee12627048a4ab5725f4768a3279), p. 225) |
| Empowerment | “a process, a mechanism by which people, organizations, and communities gain mastery over their affairs” ([[4]](#_CTVL0018aabcb7496a84bb0bde6e9dbc13f4a77), p. 122) | / |
| Engagement | “defined as a positive, fulfilling, work-related state of mind that is characterized by vigor, dedication, and absorption” ([[5]](#_CTVL001564bb70437754f63a1b03d11d5d5124f), p. 74) | Questionnaire ([[6]](#_CTVL00141be07a3526a408bb71a6ed7a5ec7388), p. 389) |
| Group effectiveness | “partnership formation, dynamics, relationships among group members, and collective action that are considered integral to the effectiveness of the group in attaining its outcomes” ([[7]](#_CTVL00124e22c4acedf4c089dadb5c21061e259), p. 150, as cited in [[8]](#_CTVL001455c4d78766346ab90895cc4cbe15eae)) | Survey instrument for evaluating group dynamics characteristics and intermediate measures of partnership effectiveness ([[7]](#_CTVL00124e22c4acedf4c089dadb5c21061e259), p. 259-261) |
| Leadership | “health promoting leadership can be defined as leadership that is concerned with creating a culture for health promoting workplaces and values to inspire and motivate the employees to participate in such a development” ([[9]](#_CTVL00179cbfb284e0541afb6c56c8b3990b03b), p. 111)  “leadership support is defined as leaders’ involvement in, and promotion of, activities, policies and practices that encourage the development of such a climate [workplace health promotion]” ([[10]](#_CTVL0013e3ef340dc1748d28d208fe1f373ccc2), p. 515) | / |
| Organizational culture | “values, beliefs and expectations shared by the members of an organisation” ([[11]](#_CTVL0010c99b2e1c3914ee2bc68132aeba259ad), p. 4, as cited in [[12](#_CTVL00183b4a90ae9234805b345ee14e3e1f81b)[–14]](#_CTVL001077f62c8e2a44d29897a2fa28f5322c2)) | Organizational Culture Inventory (OCI) ([[15]](#_CTVL0011f2235d6f68d494a881e5543d9788213), p. 149)  Survey of Organizational Culture (SOC) ([[11]](#_CTVL0010c99b2e1c3914ee2bc68132aeba259ad), p. 7) |
| Organizational readiness | “the extent to which organizational members are psychologically and behaviorally prepared to implement organizational change” ([[16]](#_CTVL00194e3d441cd884a3f9797c93471ab6395), p. 381) | Organizational Readiness for Implementing Change (ORIC) ([[17]](#_CTVL00141141042f280462d98b24a39aeddb788), Additional file 1) |
| Ownership | “the extent to which people feel involved in a programme, able to influence its direction and outcome; perceptions of the relevance of the programme to people's needs and priorities; and perceptions of the feasibility of actions which are advocated through a programme” ([[18]](#_CTVL001caf5e8d090534e7090d88cca0df5d512), p. 40) | / |
| Resources | “resources reflecting policy-makers’ individual abilities as well as the capacities of their organizations (e.g. personnel, finances)” ([[19]](#_CTVL001a0a01c245ad0454f813598aec48c3bb9), p. 324) | Quantitative questionnaire ([[20]](#_CTVL00141cb82ba3adc4cbbb8561362bf171953), p. 73) |

# References

1. O’Loughlin J, Renaud L, Richard L, Sanchez Gomez L, Paradis G. Correlates of the Sustainability of Community-Based Heart Health Promotion Interventions. Prev Med Rep. 1998;27:702–12. doi:10.1006/pmed.1998.0348.

2. Klein HJ, Molloy JC, Brinsfield CT. Reconceptualizing Workplace Commitment to Redress a Stretched Construct: Revisiting Assumptions and Removing Confounds. AMR. 2012;37:130–51. doi:10.5465/amr.2010.0018.

3. Klein HJ, Cooper JT, Molloy JC, Swanson JA. The assessment of commitment: advantages of a unidimensional, target-free approach. J Appl Psychol. 2014;99:222–38. doi:10.1037/a0034751.

4. Rappaport J. Terms of empowerment/exemplars of prevention: toward a theory for community psychology. Am J Community Psychol. 1987;15:121–48. doi:10.1007/BF00919275.

5. Schaufeli WB, Salanova M, González-romá V, Bakker AB. The Measurement of Engagement and Burnout: A Two Sample Confirmatory Factor Analytic Approach. Journal of Happiness Studies. 2002;3:71–92. doi:10.1023/A:1015630930326.

6. Torp S, Grimsmo A, Hagen S, Duran A, Gudbergsson SB. Work engagement: a practical measure for workplace health promotion? Health Promot Int. 2012;28:387–96. doi:10.1093/heapro/das022.

7. Schulz AJ, Israel BA, Lantz P. Instrument for evaluating dimensions of group dynamics within community-based participatory research partnerships. Evaluation and Program Planning. 2003;26:249–62. doi:10.1016/S0149-7189(03)00029-6.

8. Israel BA, Cummings KM, Dignan MB, Heaney CA, Perales DP, Simons-Morton BG, Zimmerman MA. Evaluation of health education programs: current assessment and future directions. Health Education Quarterly. 1995;22:364–89. doi:10.1177/109019819402200308.

9. Eriksson A, Axelsson R, Bihari Axelsson S. Development of health promoting leadership – experiences of a training programme. Health Education. 2010;110:109–24. doi:10.1108/09654281011022441.

10. Milner K, Greyling M, Goetzel R, Da Silva R, Kolbe-Alexander T, Patel D, et al. The relationship between leadership support, workplace health promotion and employee wellbeing in South Africa. Health Promot Int. 2015;30:514–22. doi:10.1093/heapro/dat064.

11. Tucker RW, McCoy WJ, Evan LC. Can Questionnaires Objectively Assess Organisational Culture? Journal of Managerial Psychology. 1990;5:4–11. doi:10.1108/02683949010000602.

12. Pettigrew AM. On studying organizational cultures. Administrative Science Quarterly. 1979;24:570. doi:10.2307/2392363.

13. Pascale R, Athos A. The art of japanese management. New York: Simon and Schuster; 1981.

14. Schein E. Organizational Culture and Leadership. San Francisco: Jossey-Bass; 1985.

15. Cooke RA, Szumal JL. Using the organizational culture inventory to understand the operating cultures of organizations. In: Ashkanasy NM, Wilderom CPM, Peterson MF, editors. Handbook of organizational culture and climate. Thousand Oaks, CA: Sage; 2000. p. 147–162.

16. Weiner BJ, Amick H, Lee S-YD. Conceptualization and measurement of organizational readiness for change: a review of the literature in health services research and other fields. Medical Care Research and Review. 2008;65:379–436. doi:10.1177/1077558708317802.

17. Shea CM, Jacobs SR, Esserman DA, Bruce K, Weiner BJ. Organizational readiness for implementing change: a psychometric assessment of a new measure. Implement Sci. 2014;9:7. doi:10.1186/1748-5908-9-7.

18. Nutbeam D. Evaluating health promotion – progress, problems and solutions. Health Promot Int. 1998;13:27–44. doi:10.1093/heapro/13.1.27.

19. Rütten A, Gelius P, Abu-Omar K. Policy development and implementation in health promotion – from theory to practice: the ADEPT model. Health Promot Int. 2010;26:322–9. doi:10.1093/heapro/daq080.

20. Rütten A, Lüschen G, von Lengerke T. Health promotion policy in Europe: rationality, impact, and evaluation. München: Oldenbourg; 2000.
